# Cryofixation of Inactivated Hantavirus-Infected Cells as a Method for Obtaining High-Quality Ultrastructural Preservation for Electron Microscopic Studies

**DOI:** 10.3389/fcimb.2020.580339

**Published:** 2020-11-06

**Authors:** Amar Parvate, Ranjan Sengupta, Evan P. Williams, Yi Xue, Yong-Kyu Chu, Robert V. Stahelin, Colleen B. Jonsson

**Affiliations:** ^1^Department of Biological Sciences, Purdue University, West Lafayette, IN, United States; ^2^Medicinal Chemistry and Molecular Pharmacology and the Purdue Institute for Inflammation, Immunology and Infectious Disease, Purdue University, West Lafayette, IN, United States; ^3^Department of Microbiology, Immunology and Biochemistry, University of Tennessee Health Science Center, Memphis, TN, United States; ^4^Center for Predictive Medicine, University of Louisville, Louisville, KY, United States

**Keywords:** hantaviruses, TEM, cryofixation, HPS-FS, virus-inactivation, membrane morphology, Hantaan virus, Andes virus

## Abstract

Hantaviruses rewire the host cell and induce extensive membrane rearrangements for their replication and the morphogenesis of the virion. Transmission electron microscopy (TEM) is a powerful technique for imaging these pathological membrane changes especially when combined with large volume electron tomography. Excellent preservation of membrane structure can be obtained when chemical fixation is combined with cryofixation via high pressure freezing making the samples amenable to serial-section tomographic reconstruction. Taking advantage of this, we have optimized a hybrid method that employs aldehyde fixation, a step that is essential for virus inactivation, followed by high-pressure freezing for ultrastructural study of Hantaan (HTN) and Andes (AND) virus infected Vero E6 cells. HTNV and ANDV are two species of the *Orthohantavirus*, from the Old and New World, respectively, and the causative agents of hemorrhagic fever with renal syndrome and hantavirus pulmonary syndrome in humans. We applied the method for the qualitative assessment of the perturbation of the endomembrane system induced by HTNV and ANDV in infected vs. mock-infected cells. Screening of serial-sections revealed consistency of membrane preservation across large volumes indicating potential of these samples for tomographic studies. Images revealed large-scale perturbations of the endomembrane system following HTNV-infection that included the dilation of the rough endoplasmic reticulum and fragmentation of the Golgi apparatus. Infected cells exhibited a tendency to accumulate large numbers of vacuoles that were especially apparent in ANDV. In summary, our hybrid method provides a path for the study of BSL-3 pathogens using cutting edge 3D-imaging technologies.

## Introduction

Sample preparation for transmission electron microscopy (TEM) analysis has classically involved chemical fixation in combination with alcohol dehydration at room temperature. Chemical fixation is essential to stabilize cellular architecture against down-stream processing steps and has been shown in many cases to preserve the cellular architecture (Huang and Yeung, 2015). However, chemical fixatives such as aldehydes are known to be poor at preventing extraction of lipids or complex carbohydrates at the dehydration and staining step (Zechmann et al., 2007).

Cryo-fixation via high pressure freezing has been shown to minimize these disruptive changes during sample preparation (Sosinsky et al., 2008; Mielanczyk et al., 2014). Cryo-fixation is a form of physical fixation that provides excellent preservation of the cellular ultrastructure when performed under high-pressure as it minimizes disruptive ice crystal formation (McDonald and Auer, 2006). As a result, HPF has become readily employed for TEM tomography research and is employed in conjunction with freeze-substitution (FS) to prepare samples for room temperature, resin-based TEM analysis. HPF is particularly advantageous for obtaining projection images or tomographic data imaging which rely on sections of 0.1–0.5 μm thicknesses (Buser and Walther, 2008). However, instruments like the Leica EM PACT (for HPF), Leica EM AFS (for FS) and an ultramicrotome used to prepare samples for electron tomography are expensive and rarely found in individual laboratories.

Many viruses remodel cellular organelles to create neo-organelles for replication and egress (Novoa et al., 2005; Miller and Krijnse-Locker, 2008). Additionally, reorganization of intermediate filaments such as vimentin have been implicated as a structural component in the replication processes of several viruses [for examples see (Stefanovic et al., 2005; Kanlaya et al., 2010; Ghosh et al., 2011; Gladue et al., 2013)]. For example, we have previously shown the Hantan virus (HTNV) induces vimentin reorganization in Vero E6 (Ramanathan and Jonsson, 2008). However, due to the unavailability of HPF and FS within the confines of most high containment biosafety facilities, the application of advanced imaging techniques to the study of the intracellular compartments that require good ultrastructural preservation have lagged. Thus, TEM studies of such deadly pathogens have mostly been carried out using traditional chemical and dehydration techniques that occasionally result in poor preservation and artifacts, which renders the samples unsuitable for high resolution tomographic studies.

Herein, we explore the application of advanced sample preparation methodology to the study of viruses within the genus *Orthohantavirus*. TEM analysis has been extensively reported for the Old World prototypic member, HTNV and a few New World viruses using conventional approaches (Lee, 1981; Ravkov et al., 1997; Xu et al., 2007). However, these samples provide a limited view into the virus induced cellular modification and are further not suitable for tomography. Using HPF-FS to obtain images for 2D and tomographic data collection is a promising approach to study hantaviruses (Venter et al., 2013). However, it is extremely challenging to set up the equipment needed to carry out HPF and FS inside a BSL-3 facility (Sherman et al., 2013). Secondly, cells infected with BSL-3 viruses need to be completely inactivated if they are to be removed from the BSL-3 facility. In the absence of a protocol to facilitate HPF and FS inside a BSL-3 facility, researchers have continued to obtain images and study intracellular replication of hantaviruses by preparing samples through conventional fixation.

Hybrid methods that employ aldehyde fixation in combination with high pressure freezing and staining via freeze substitution have been shown to preserve tissues better than only chemical fixation in combination with alcohol dehydration (Sosinsky et al., 2008). In many cases, the sample preservation is even comparable to live, unfixed high pressure frozen cells (Walther and Ziegler, 2002; Buser and Walther, 2008). This was shown to be true even when it was used in the subcellular localization of APEX2- and horse radish-peroxidase tagged cellular and bacterial proteins (Sengupta et al., 2019; Herrera et al., 2020; Ward et al., 2020). Additionally, Venter et al. (2013) demonstrated that even in the case of prolonged storage in glutaraldehyde and subsequent HPF-FS retained excellent ultrastructural integrity in comparison with conventional sample processing. This feature of the hybrid method fits perfectly with the study of BSL-3 pathogens as infected cells need prolonged exposure to aldehyde fixatives for viral inactivation prior to bringing the sample outside the BSL-3 for further processing and imaging. Indeed, this knowledge has been utilized for studying the biogenesis of double membrane vesicles in HCV infected Huh7 cells (Romero-Brey et al., 2012).

Here, we have optimized the hybrid approach of fixation with extended freeze substitution for an ultrastructural study of Old and New World Hantavirus infected cells. The hybrid method fits the necessity for the chemical inactivation of virus prior to removal of infected cells from the BSL-3 laboratory for high-pressure freezing and freeze substitution. Our method enabled the study of hantavirus-infected cells outside a BSL-3 lab to enable high quality images. The ability to follow large subcellular structures through several sections showcases the consistency of membrane preservation achieved in this approach.

## Materials and Methods

### Cells, Viruses and Inactivation of Virus-Infected Cells

Vero E6 cells were propagated in Dulbecco's modification of Eagle's Medium (DMEM) supplemented with heat-inactivated 10% fetal bovine serum (FBS) (Sigma-Aldrich) at 37°C with 5% CO_2_. Cells were mock-infected or infected with HTNV strain 76–118 or ANDV strain Chile-9717869 at a MOI of 0.1. HTNV and ANDV were provided by Dr. Connie Schmaljohn (USAMRIID, Frederick, MD, USA). All infections were performed within BSL-3 containment. Cells were harvested on days 3, 7, and 9 and post-infection (DPI). Cells were detached from the flask using 0.25% trypsin for 2–3 min, resuspended with DMEM containing 10% FBS and centrifuged at 500 × *g* for 5 min at 4°C. Cells were resuspended in 0.1 M cacodylate buffer, pH 7.4 and again resuspended in 2% glutaraldehyde in 0.1 M cacodylate buffer, pH 7.4 containing 2% sucrose for 30 min on ice. Next, the infected cells were washed twice using 0.1 M cacodylate buffer to remove excess glutaraldehyde. All centrifugation steps were performed at 500 × *g* for 5 min at 4°C. Cells were resuspended in 500 μL of a cryoprotecting media (15% BSA in DMEM).

To confirm inactivation of cells prior to removal from the BSL-3 facility, we infected Vero E6 cells grown in a six well-plate with HTNV and cultured the virus for 7 days. This is a similar test used for the virus-infected supernatant (Parvate et al., 2019). Once the media was confirmed to contain no infectious virus, the cell pellets were removed from the BSL-3 facility. Supernatant was removed and the virus infected cell monolayers were washed with sterile PBS and 1% glutaraldehyde for 30 min. Treated cell monolayers were scraped off using a cell scraper and collected into a microfuge tube. Inactivated cells were sonicated to disrupt cells and then clarified with centrifugation. The sonicated cell suspensions were diluted with 10-fold serial dilution in the DMEM cell culture media followed by inoculation and cultivation onto the E6 cells grown on 96 well-tissue culture plate for 7 days. As a control untreated virus culture media and a sonicated cells from untreated virus grown cells. A week later inoculated cells were fixed with Acetone-Methanol (1:1) mixture and air-dried followed by ELISA using a polyclonal immune serum to detect hantavirus growth. All inactivated samples have shown negative result, but positive control samples of untreated sonicated virus cultured cells showed 1 × 10e5 virus.

### High-Pressure Freezing and Freeze-Substitution (HPF-FS)

The HTNV or ANDV-infected or mock-infected Vero E6 cells in the 15% BSA-DMEM were frozen as pellets on gold 1.5 × 0.5 mm membrane carriers (NCI) using the EM PACT2 high-pressure freezer (Leica). Freeze substitution of the frozen cells was carried out by an AFS2 system (Leica) using 0.25% tannic acid and 5% ddH_2_O in acetone at −90°C for 24 h. Next, the cells were washed with cold (−90°C) anhydrous acetone three times and exposed to 0.5% uranyl acetate, 3% osmium tetroxide, and 5% ddH_2_O in acetone. The temperature was increased to −80°C, where it was held for 72 h. The temperature was then increased by 10°C/h increments and held at −20°C for 6 h. Finally, the cells were warmed to 0°C by the same increments followed by washing three times with cold (0°C) acetone. Infiltration was done with gradual increasing concentration of Durcupan resin (Sigma-Aldrich) in acetone (2, 4, 8, 16, 25, 75, and 100% without the presence of the resin activator for 4 h each). A final embedding in 100% Durcupan with activator C was done at 60°C for 48 h to polymerize the resin. Total time taken for this procedure was 154 h.

### Transmission Electron Microscopy

Thin sections (90 nm) were obtained from resin-embedded (Durcupan, Sigma-Aldrich) blocks containing HTNV, ANDV or mock-infected Vero E6 cells using a Leica EM UC7 ultramicrotome. Serial-sections were collected on formvar-coated 2 × 1 mm copper slot grids (Electron Microscopy Sciences). Sample grids were then post-stained with 2% uranyl acetate followed by Sato's lead solution. The stained sections were screened using TEM at 80kV (FEI, Tecnai, T-12) or at 200 kV (Phillips, CM200) mounted with Gatan 4K or a 2K camera, respectively, at Purdue University. Images were examined for their quality of the cellular ultrastructural preservation and to assess staining of organelle structure using ImageJ.

## Results

### Optimization of the High Pressure-Freeze Substitution Protocol for Glutaraldehyde-Fixed Vero E6 Cells

**Figure 1:**
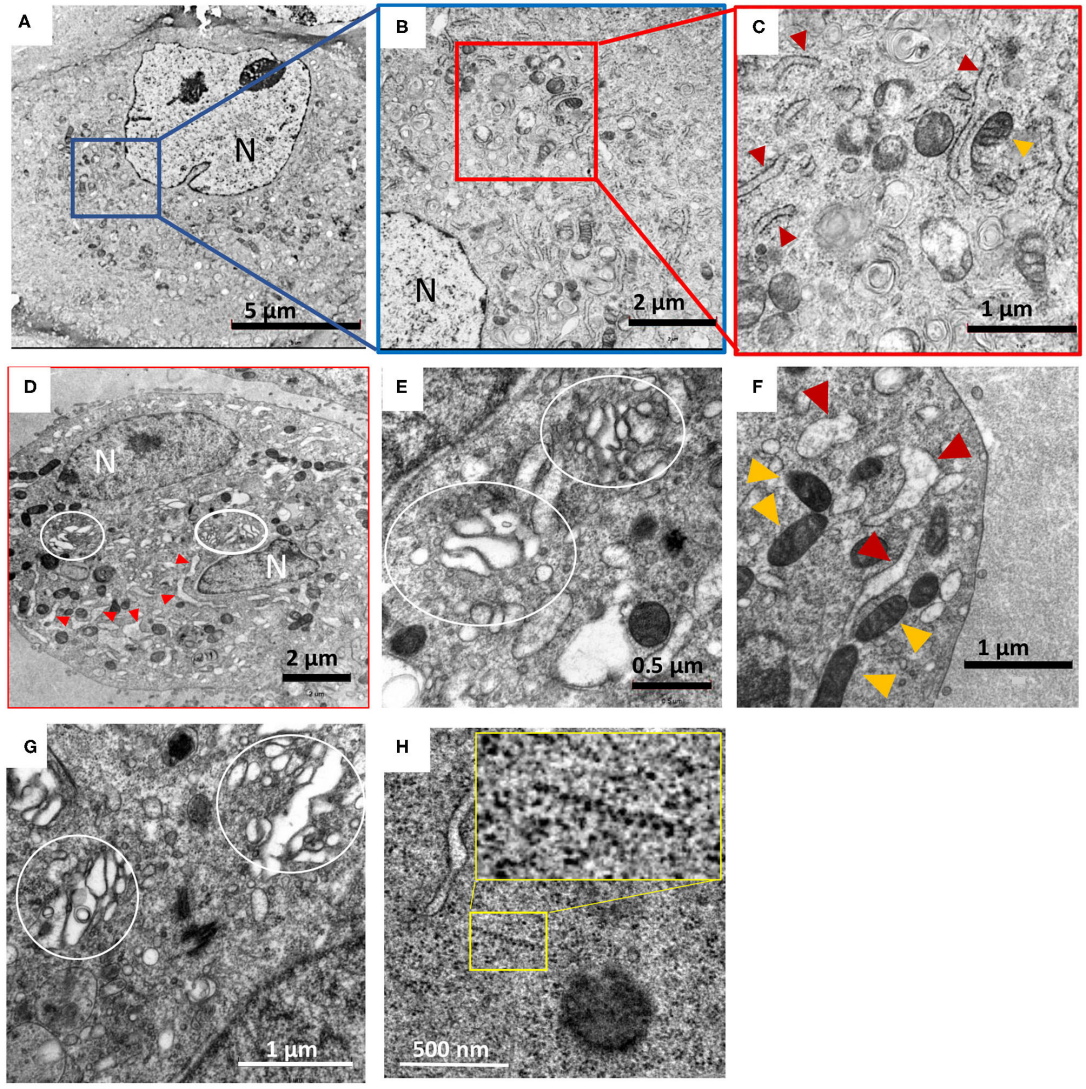
Hybrid chemical-cryo fixation of cells in conjunction with extended freeze substitution imparts excellent ultrastructural preservation of HTNV infected Vero E6 cells. Images **(A)** through **(D)** were collected at 5,000–6,000 X magnification and **(E)** through **(H)** were collected at 13,500–25,000 X magnification. **(A,B,C)** are images of mock infected Vero E6 cell, processed by HPF-extended FS (ex-FS) method. **(D–H)** Show changes in cells due to hantavirus infection. N, indicates nucleus, with red and orange triangles denoting rough ER and mitochondria, respectively. **(D,E)** HTNV infected cells at 7 days post infection exhibit distended and fragmented Golgi apparatus (marked by white circles). **(F)** Swelling of the rough ER is widespread and is a hallmark of HTNV infection along with Golgi unstacking and fragmentation **(E,G)**. **(H)** Typical features of HTNV infection such as distended Golgi apparatus and wide-spread polyribosomes [yellow box and magnification of the area within the box in inset, **(H)**] were also observed. The term distended refers to the swelling and loss of tight Pan cake like stacked morphology we noted similar to that reported by others (Salanueva et al., 2003; Lazaro-Dieguez et al., 2006).

One can often follow the progression of viral infections over time in mammalian cells via TEM by tracking of immature or mature virions or tracking virus-induced membrane modifications (Beachboard et al., 2015). Thus, preservation of cell samples and integrity of the cellular membranes is of paramount importance if the samples will be examined using electron tomography. Hybrid methods have been used successfully where aldehyde fixation is combined with HPF-FS to provide superior quality staining and preservation of cellular membranes. Hence, our protocol was initially optimized on mock-infected Vero E6 cells, which were fixed with 2% glutaraldehyde (Figures 1A–C). After HPF, several combinations of substitution protocols were tested along with different concentrations of UA, OS, and tannic acid (TA). First, we assessed the preservation and membrane staining of cell pellets prefixed with glutaraldehyde using a short 72 h protocol typically used for staining and organic substitution of cryofixed live cells. This resulted in poor membrane staining and preservation of cells. Integrity of the cellular membranes and sample preservation of the mock infected samples was analyzed by TEM and was found similar to the images reported in literature (Goldsmith et al., 1995; Ravkov et al., 1997; Xu et al., 2007).

We also used an *extended-FS protocol* where cells were immersed in osmium tetroxide and uranyl acetate for 72 h at −80°C and was ramped up to 0°C slowly over a period of 2 days (Supplementary Table 1). This approach resulted in excellent preservation of the cell cytoplasm and membrane as shown in representative images (Figures 1A–C). Preservation was assessed based on several criteria including membrane integrity, dense packing of the cytoplasm and maintenance of organelle structure. Smoothness of intracellular membrane and integrity of the nuclear envelope is often use as a yardstick for good cellular preservation (Sosinsky et al., 2008; Tsang et al., 2018; Sengupta et al., 2019). Our method indeed yielded smooth, continuous nuclear envelope that exhibited uniform distance between the membrane bilayer confirming good membrane preservation. Within the nucleoplasm, the euchromatin and heterochromatin can also be distinguished by their differential staining (Supplementary Figures 1, 2). This optimized protocol was then used to process hantavirus-infected cells.

### Preparation and Analyses of HTNV-Infected Vero E6 Cells by HPF and an Extended-FS (HPF-ex-FS) Protocol for TEM

HTNV-infected Vero E6 cells were harvested at 3, 7, and 9 dpi. Mock-infected control cells were also harvested to differentiate between non-infection induced stress and virus induced stress in the cells after the duration of infection. After fixation and confirmation of inactivation, HTNV-infected Vero E6 cells were removed from BSL-3 and processed using the HPF-ex-FS protocol (Sengupta et al., 2019).

Mock and HTNV-infected cells showed similar staining and membrane preservation and integrity of cellular organelles (Figures 1D–F) and were comparable to the images obtained in the initial round of optimization (Figures 1A–C). At 3 dpi, HTNV-infected cells showed no visible membrane perturbations and were comparable to uninfected controls. Other changes in intracellular morphology included widespread herniation of the rough ER and the Golgi and loss of typical stacked Golgi architecture after 7 dpi (Figures 1D,E) and fragmentation of the Golgi apparatus was widespread. It was observed that even after 9 days, the mock-infected cells had fewer vacuoles compared to HTNV-infected cells. Even after 9 dpi, no inclusion bodies were observed in any of our samples, although loss of typical stacked cisternal architecture of the Golgi and fragmentation was quite widespread (Figure 1G). Polyribosomes were also observed in the cytosol indicating a high amount of viral protein translation (Figure 1H).

**Figure 2:**
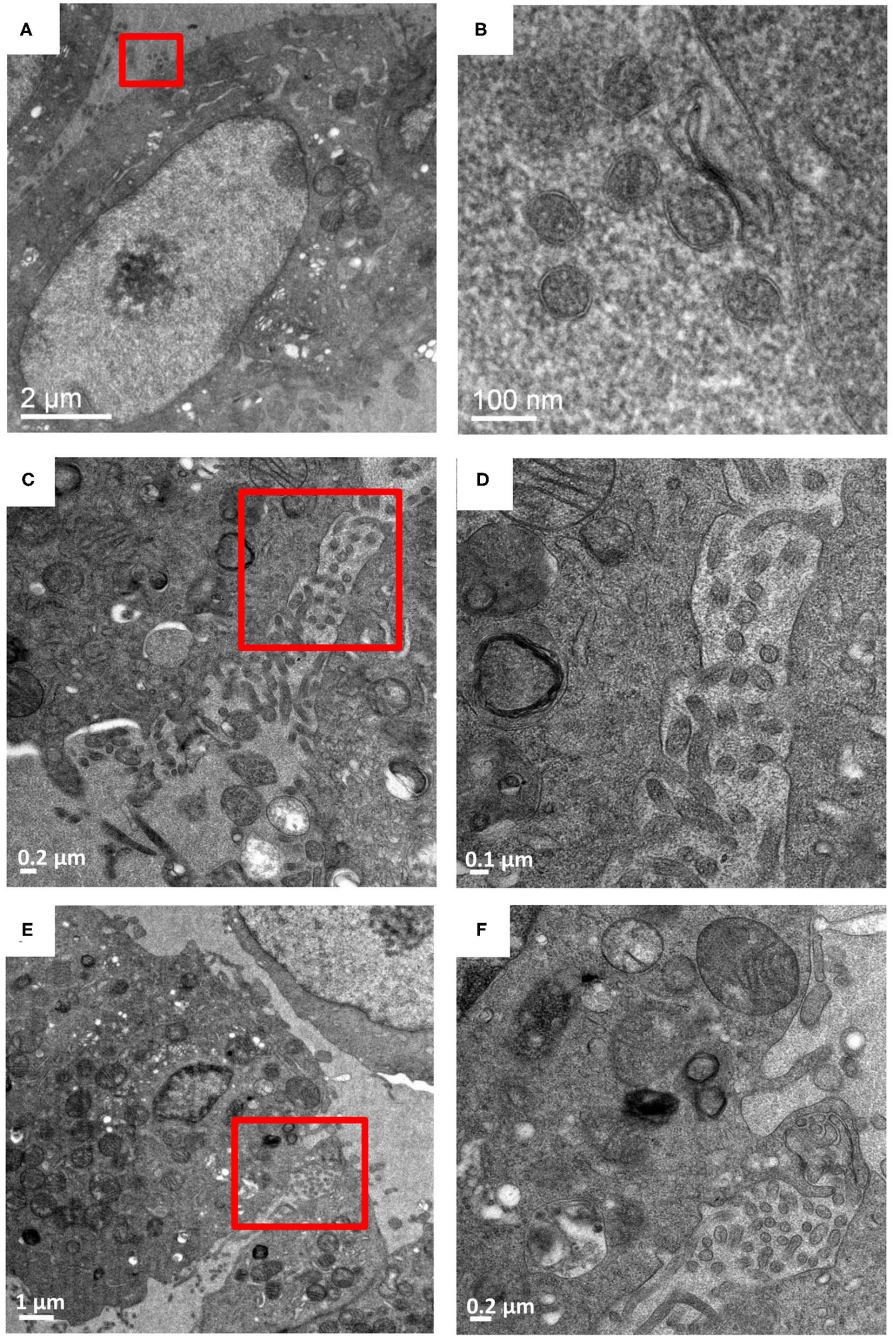
Budded Hantaan virions between adjacent cells. Vero cells infected with HTNV imaged at 9 dpi. **(A,C,E)** are images at 5,000–6,000 X magnification with **(B,D,F)** as corresponding images of the regions show in in the red boxes at 13,500–26,000 X magnification. **(A,B)** Virions measuring 70–90 nm exterior to the plasma membrane. **(C,D)** virions between two adjacent cells. **(E,F)** show virions between two adjacent cells sequestered by plasma membrane projections from the cells.

Extracellular HTN virus-like-particles were observed in samples screened at 7 and 9 dpi, but not at 3 dpi (Figure 2, Supplementary Figure 3). Extracellular virus-like particles were more numerous in the area between adjacent cells (Figures 2A–D). No intracellular viruses were observed at 3 dpi. The virus-like particles displayed overall round and pleomorphic morphology. The lipid bilayer envelope of the particles could easily be distinguished in the images (Figure 2B) but no glycoproteins were visible. Dimensions of about 100 such particles were measured using ImageJ and estimated to be 90 ± 20 nm. The overall smaller diameter of HTN virions observed in cellular samples relative to purified virions (Parvate et al., 2019) could be attributed to “shrinking” of the resin sections in the electron beam (Kizilyaprak et al., 2015). Plasma membrane projections from the infected cells were also observed to have virions near them that seemed to sequester egressing virions (Figures 2E,F). These projections were not observed in control cells and were inferred to be a virus induced membrane modification.

### HTNV Sequestering by Plasma Membrane Projections

**Figure 3:**
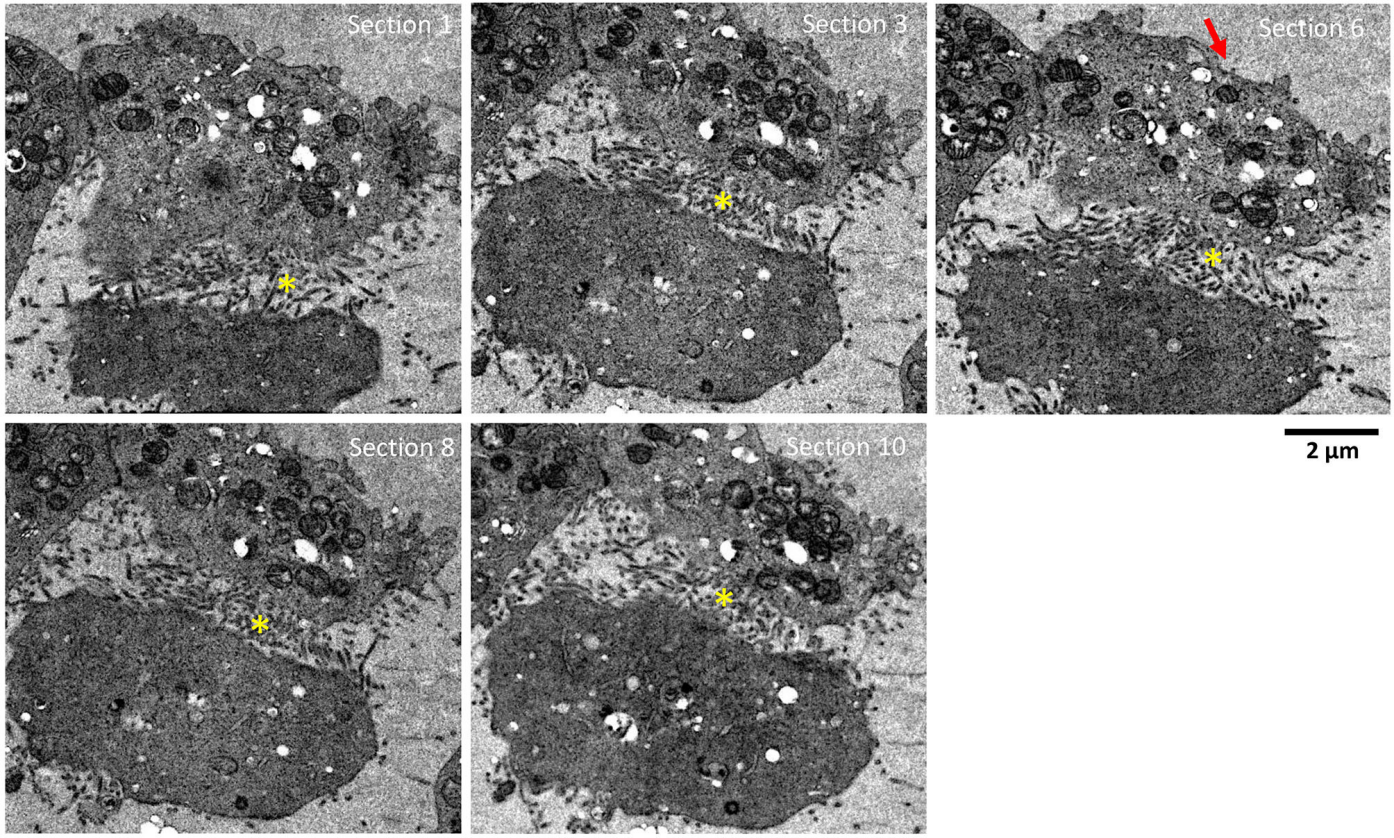
Serial sections showing adjacent HTNV-infected Vero E6 cells. Serial sections, 90 nm thick, through HTNV-infected Vero E6 cell at 9 dpi. Successive sections show lots of projections only in areas between neighboring infected cells. These sections are visible in multiple cells in contact. The depth of sample covered was about 1 μm. Yellow (*) indicates the area between two cells with multiple plasma membrane projection contacts from both cells putatively sequestering viruses. Red arrow indicates area of cells not in contact with adjacent cells and devoid of plasma membrane projections.

To further screen for plasma membrane projections (i.e., extracellular sequestering virus-like particles), serial sections of the same two adjacent HTNV-infected Vero E6 cells were tracked and imaged for 10 sections of 90 nm each (Figure 3, Supplementary Figure 4). This corresponded to a depth of ~1 μm in the cell. Membrane projections were found only in regions where the two cells were juxtaposed. While these projections were observed throughout the depth of the cell imaged, they were not observed in control cells. Similar structures have been documented in *Orthobunyavirus* where the plasma membrane projections were found to harbor egressing virions (Sanz-Sanchez and Risco, 2013). While we observed similar plasma membrane projections in HTN infected cells, a combination of immunochemical studies and tomographic analyses would be needed to confirm whether these projections harbor HTN virions or not.

### Preparation and Analyses of ANDV-Infected Vero E6 Cells by HPF-ex-FS Protocol for TEM Imaging

**Figure 4:**
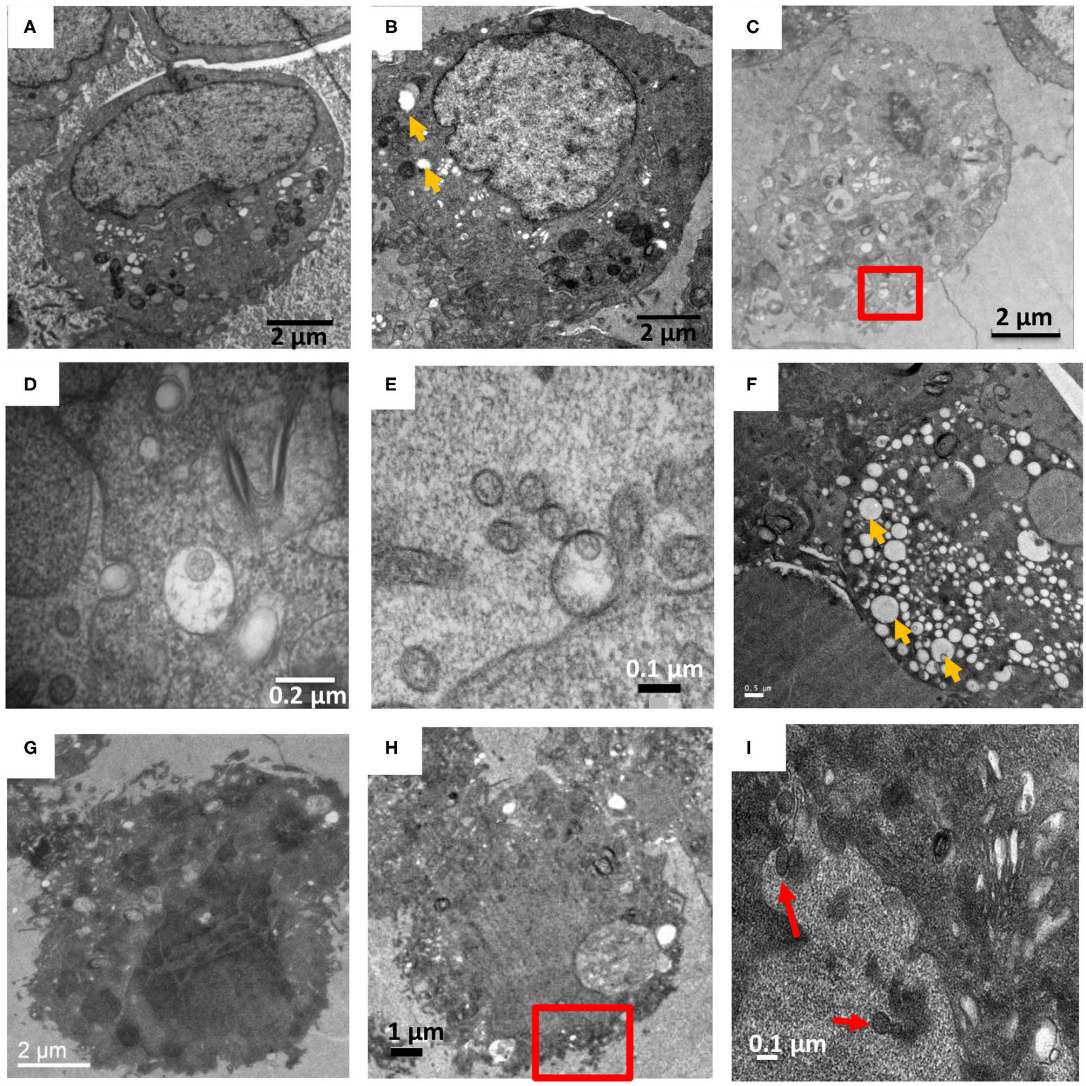
HTNV and ANDV infection of Vero E6 on 7 and 9 dpi. **(A–E)** are HTNV infected cells. **(A,B)** at 7 and 9 dpi respectively, where cells show relatively few vacuoles (orange arrow). **(C)** At 9 dpi, a cell is shown with herniated ER and fragmented Golgi with the red square indicating a virus like particle in a vesicle close to the plasma membrane. Other HTN virions also seen external to the plasma membrane. **(D)** Zoomed in view of the red square in **(C)**. **(E)** A 9 dpi timepoint showed a single virus particle enclosed inside a vesicle external to the plasma membrane along with other extracellular virus like particles. **(F–I)** ANDV infected VeroE6 cells. **(F)** On 7 dpi, a cell showed a high degree of vacuolation and stress as compared to **(A)**. **(G)** On 9 dpi, a cell showed very dark staining indicating that the cell may have been lysed. No organelles were discernable. **(H)** Low magnification image of 9 dpi with red box indicating AND virus-like particles external to the plasma membrane. **(I)** Zoomed in view of area marked in **(H)** showing pleomorphic virions external to the plasma membrane. Red arrow indicates extracellular ANDV.

To compare how New and Old World hantavirus infection induced morphological changes in Vero E6 cells, ANDV-infected Vero E6 cells were harvested and processed on 7 and 9 dpi (Figures 4E–H). For every timepoint, several grids worth of sections were collected and for every grid, at least 4–5 cells were observed across 5–10 sections to track the virus induced changes in the cell. These images were compared to the HTNV-infected Vero E6 cells from the same timepoint (Figures 4A–D). In the representative images of HTNV-infected cells, well-preserved and intact cellular membranes were visible on 7 and 9 dpi (Figures 4A,B), but intracellular virions were rarely observed (Figures 4C,D). We observed one HTN virus like particle in separate vesicles internal and external to the plasma membrane (Figures 4D,E, Supplementary Figure 4) although several virus-like particles were not enclosed by any vesicle external to the plasma membrane (Figures 2C,F, Supplementary Figure 2). In contrast, the ANDV-infected cells looked extremely stressed and highly vacuolated even at 7 dpi (Figures 4F,G). These cells stained very dark throughout the section and no intracellular membranes or organelles could be distinguished (Figures 4G,H). The cells for the 9 dpi timepoint looked lysed or close to death, which was very different from HTNV-infected cells (Figures 3A,B).

Due to the high number of vacuoles and the dark staining obtained for ANDV infected cells, we couldn't observe intracellular particles nor any virus sequestration between adjacent cells. However, some virus-like particles were observed external to the plasma membrane (Figures 4H,I). A single vesicle comprising multiple pleomorphic ANDV virions was also observed but it was not in proximity to any cell on that section (Supplementary Figure 5).

## Discussion

Analyses of TEM images processed using the method reported herein revealed large scale changes in the endomembrane system following HTNV-infection that included the dilation of the rough endoplasmic reticulum and unstacking and vesiculation of the Golgi apparatus (Figures 1D,E, Supplementary Figures 1, 2). HTNV assembles at the Golgi, so at some point the assembled RNPs must traffic to the Golgi. Virions have been visualized by electron microscopy in this compartment (Tao et al., 1987). The RNP presumably buds into the Golgi to produce the virion, and then the virion exits the Golgi through the formation of a vesicle surrounding the hantavirus particles (Schmaljohn et al., 1986). We observed tubular projections and budding at the plasma membrane with virus accumulation in the extracellular space at 7 and 9 dpi. The New World hantaviruses are proposed to bud from the plasma membrane (Goldsmith et al., 1995).

Based on visual observations, while HTNV infected cells showed presence of vacuoles at 7 and 9 DPI, vacuolization was more extensive in the ANDV infected cells. Prior TEM studies on the analysis of virus-mediated cellular changes used high MOIs from 5 to 20 (Ravkov et al., 1997). Cells harvested in this study were infected at a MOI of 0.1. In our studies, we also collected the supernatant on day 7 from both ANDV and HTNV infected cells to prepare inactivated purified virus samples for cryo-EM studies and cryo-EM images of intact virions were obtained from both samples (Parvate et al., 2019). Additionally, Vero E6 cells used as controls did not show such extensive vacuolation or dark staining of the cytosol at 7 or 9 dpi. This suggests that our observations of the highly vacuolated cells are a result of the ANDV infection.

To date, TEM based ultrastructural studies of cells infected with hantaviruses have employed chemical fixation in combination with alcohol dehydration, a combination that is sometimes prone to extraction of lipids leading to poor preservation of membrane and the cytosol. In some cases, this could be highly disruptive to subcellular membrane structures resulting in artifacts (Xu et al., 2007). These reports provide only a limited view of the subcellular structures in terms of volume of the actual cell covered since the sections are typically 60–90 nm while the thickness of a cell even on a grid is several microns. In contrast, we were able to follow cells through a thickness up to ~1 μm by following the serially collected sections. While the fixation preserves cellular integrity, the glutaraldehyde will extensively crosslink cellular proteins. With the exception of some lipids that contain primary amines like phosphatidylserine, glutaraldehyde is not known to react with lipids. One current theory is that the membranes get locked into a matrix of crosslinked proteins and this may render them difficult to stain the membranes in the short FS protocol (Migneault et al., 2004). We did observe subpar staining on mock infected control samples after a 3 day FS protocol. To circumvent this issue, we tested −80°C FS from 24 h up to 96 h. We found that 72 and 96 h resulted in comparable staining and leaving the samples in the FS mixture at room temperature following the substitution reaction, led to further improvement of the membrane staining (Romero-Brey et al., 2012). The combination of HPF and ex-FS employed in our study allowed for excellent preservation of cellular membrane in hantavirus-infected cells.

Preparing hantavirus-infected samples by our method was a lengthy process. From growing cells to imaging, a single round can take as long as 2–4 weeks depending on the length of infection and duration of FS protocol. However, our method is geared toward samples which we suggest will be good candidates for future electron tomographic studies. Our method can be combined with section TEM tomography or scanning electron microscopy or in conjunction with focus ion-beam milling (FIB/SEM). Ideally, TEM and FIB/SEM based tomographic analysis of virus-infected cells is an excellent option to study virus induced membrane reorganization. The sections of the reconstructed data can be stitched together to obtain a holistic picture of the virus induced changes by covering large volumes within the infected cell (Romero-Brey and Bartenschlager, 2015). Tomographic data will help establish structurally how, for example, the plasma membrane projections may interact with the egressing particles.

## Data Availability Statement

The raw data supporting the conclusions of this article will be made available by the authors, without undue reservation.

## Author Contributions

AP, RS, and CJ contributed in design and concept of study. AP and RS optimized the method. AP, EW, and CJ performed wet experiments. AP, RS, and YX performed TEM analyses. AP, RS, EW, and CJ were involved in manuscript writing. CJ and RVS provided supervision and funding. All authors contributed to manuscript editing and revision. All authors have read and approved the submitted version.

## Conflict of Interest

The authors declare that the research was conducted in the absence of any commercial or financial relationships that could be construed as a potential conflict of interest.
