# Supplementary material for: Cryofixation of Inactivated Hantavirus-Infected Cells as a Method for Obtaining High-Quality Ultrastructural Preservation for Electron Microscopic Studies
Source: Front Cell Infect Microbiol. 2020 Nov 6;10:580339. doi: 10.3389/fcimb.2020.580339 (PMC7677528; doi:10.3389/fcimb.2020.580339)
Supplement: Supplementary file 1 [file Table_1.docx]

Supplementary Material

# Supplementary Data

## Supplementary Figures and Tables

**Table S1.** Steps for the extended FS protocol. Total length of the protocol is 154 h.

| Start (^0^C) | End (^0^C) | Slope | Hours |  |
| --- | --- | --- | --- | --- |
| -90 | -90 | 0 | 24 | Wash 3x with cold acetone |
| -90 | -80 | 10 | 1 |  |
| -80 | -80 | 0 | 72 |  |
| -80 | -20 | 10 | 6 |  |
| -20 | -20 | 0 | 24 |  |
| -20 | 0 | 10 | 2 |  |
| 0 | 0 | 0 | 24 | Move samples to RT |
| RT | RT | 0 | 0.5-1 | Wash 3x with acetone at RT |


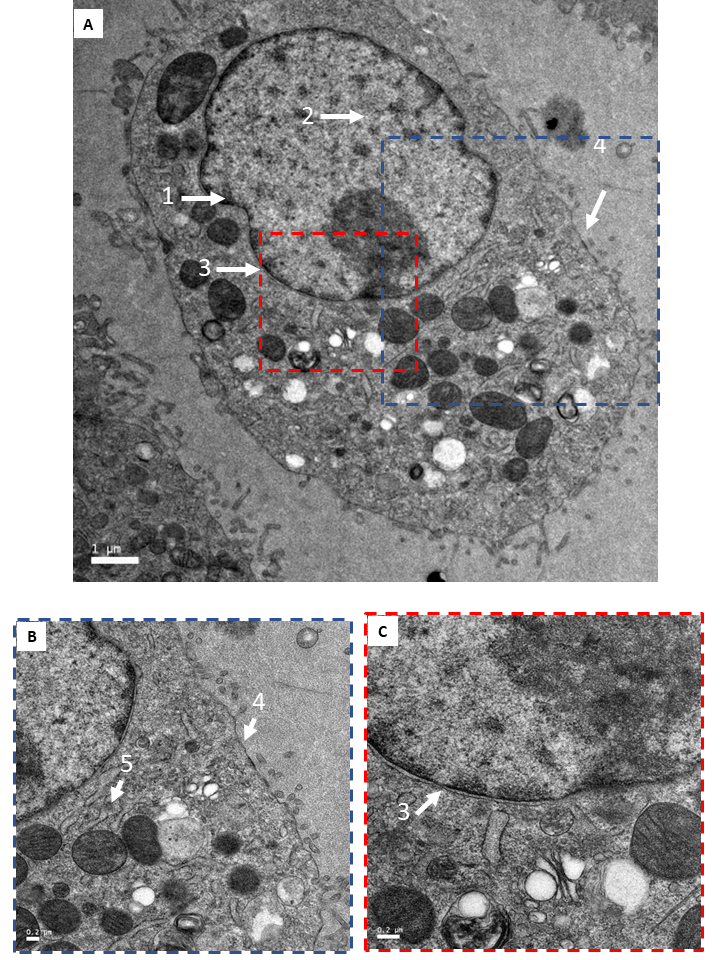


Figure S1. Preservation of cellular features post ex-FS protocol. (A) First representative Vero E6 cell at 9 DPI timepoint imaged at 5000 X magnification. Good quality of preservation and staining is indicated by 5 features (numbers and white arrows) - 1. Heterochromatin 2. Euchromatin 3. Nuclear envelope 4. Plasma membrane. 5) Smooth ER (B) Area within the blue boundary imaged at 10,000 X magnification. The plasma membrane (4) and smooth ER (5) are visible in the cell interior. (C) Area within the red boundary imaged at 13,500 X magnification. The nuclear envelope (3) is visualized in this image indicated by the arrow


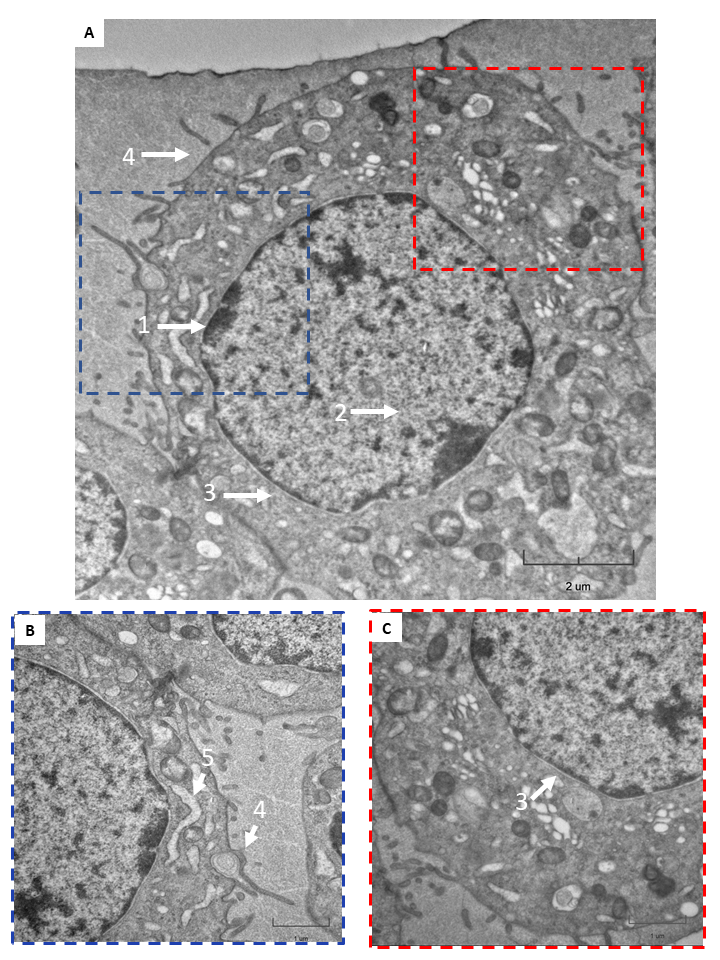


Figure S2. Preservation of cellular features post ex-FS protocol. (A) Second representative Vero E6 cell at 9 DPI timepoint imaged at 5000 X magnification. Good quality of preservation and staining is indicated by 5 features (numbers and white arrows) - 1. Heterochromatin 2. Euchromatin 3. Nuclear envelope 4. Plasma membrane. (B) Area within the blue boundary imaged at 10,000 X magnification. The plasma membrane (4) and dilated smooth ER (5) are visible in the cell interior (C) Area within the red boundary imaged at 13,500 X magnification. The nuclear envelope (3) is visualized in this image indicated by the arrow.

**
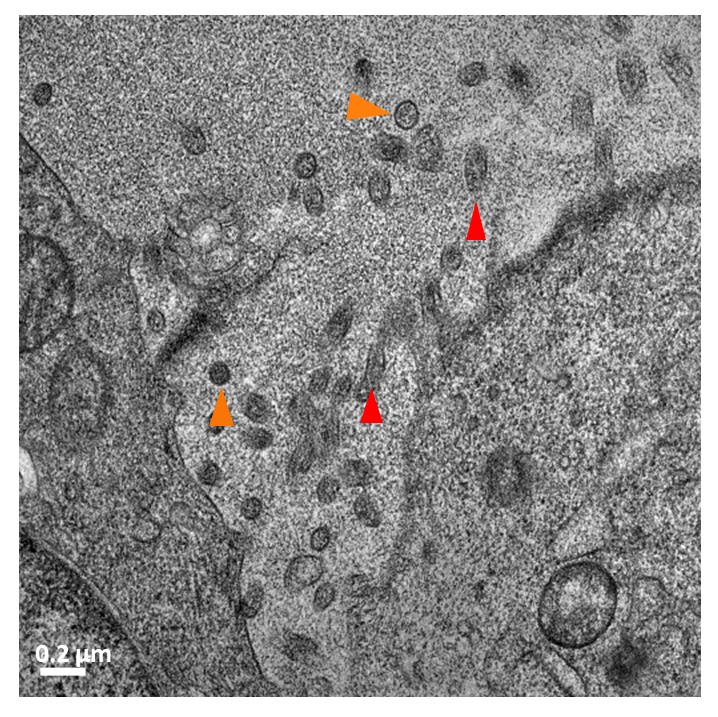
**

Figure S3. Polymorphic HTN virions. Image collected at 10,000X. A 90 nm thick section collected at 7 DPI section showing virus-like particles egressed from 2 different cells external to the plasma membrane. Most virus-like particles show a round morphology (orange triangles) while some virus-like particles (red triangles) have tubular morphology, similar to our cryo-EM images reported previously (Parvate et al., 2019).


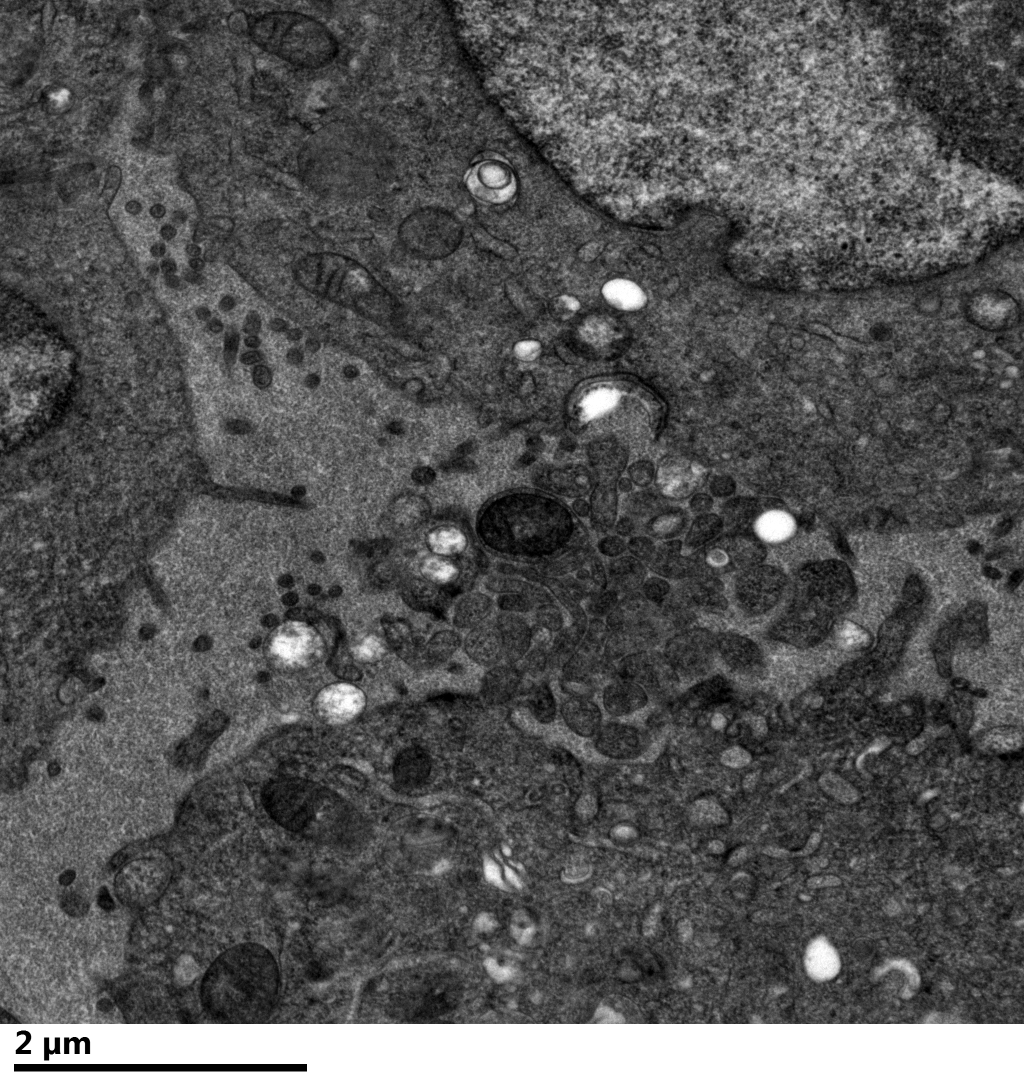


Figure S4. Extracellular HTN virus-like particles . Image collected at 5000X. A 90 nm thick section collected at 9 DPI showed several HTN virus-like particles are sequestered between juxtaposition of 3 adjacent cells.


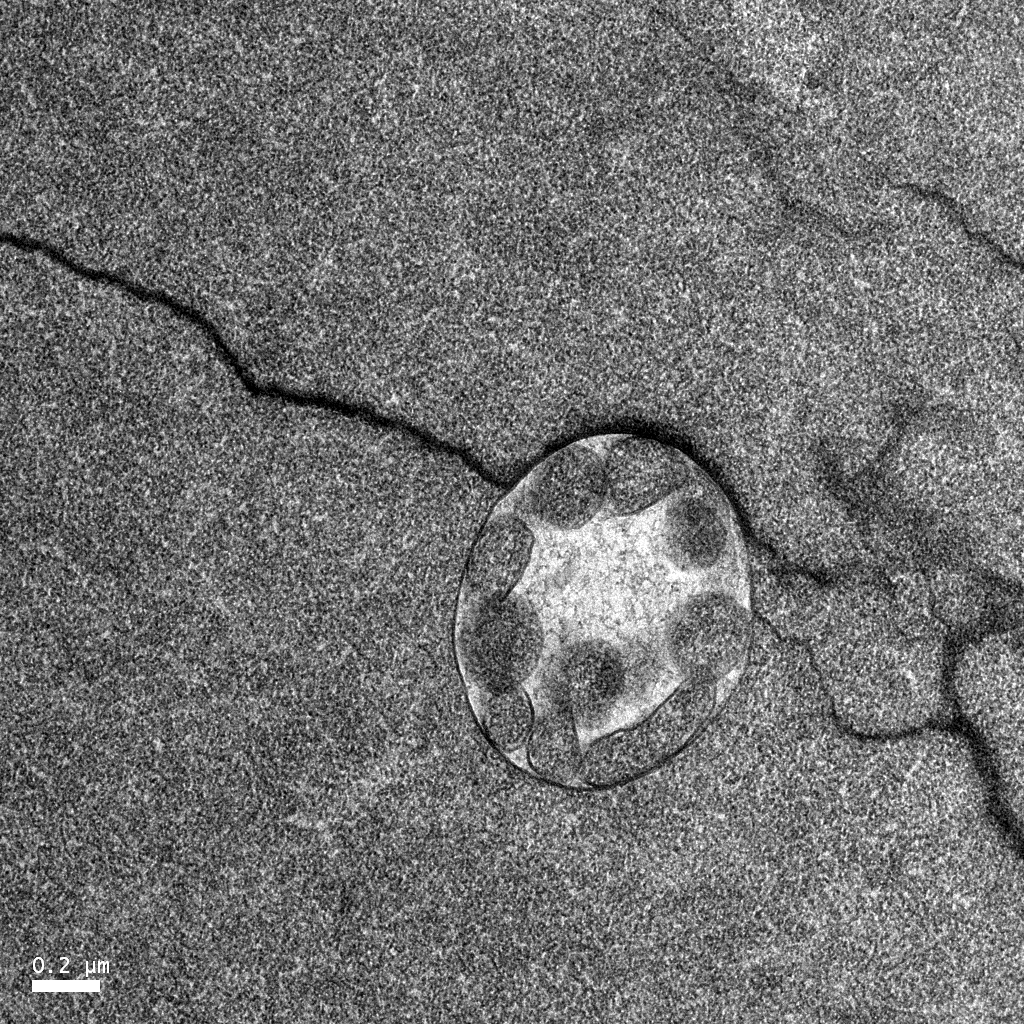


Figure S5. Vacuole filled with AND virus-like particles. Image collected at 13,500 X. A 90 nm thick section collected at 9 DPI showed a single vesicle filled with AND virus-like particles. The vesicle was not close to any cell and could not be traced in preceding or subsequent sections. However, the particles are pleomorphic and display round and tubular morphology which is similar to our cryo-EM images reported previously (Parvate et al 2019).

**References**

Parvate, A., Williams, E.P., Taylor, M.K., Chu, Y.K., Lanman, J., Saphire, E.O., et al. (2019). Diverse Morphology and Structural Features of Old and New World Hantaviruses. *Viruses* 11**,** 11. doi: 10.3390/v11090862.
